# The Effect of Focal Damage to the Right Medial Posterior Cerebellum on Word and Sentence Comprehension and Production

**DOI:** 10.3389/fnhum.2021.664650

**Published:** 2021-05-20

**Authors:** Sharon Geva, Letitia M. Schneider, Sophie Roberts, David W. Green, Cathy J. Price

**Affiliations:** ^1^Wellcome Centre for Human Neuroimaging, University College London, London, United Kingdom; ^2^Department of Cognition, Emotion and Methods in Psychology, Faculty of Psychology, University of Vienna, Vienna, Austria; ^3^Department of Experimental Psychology, Faculty of Brain Sciences, University College London, London, United Kingdom

**Keywords:** cerebellum, verbal fluency, word processing, sentence processing, lobule IX

## Abstract

Functional imaging studies of neurologically intact adults have demonstrated that the right posterior cerebellum is activated during verb generation, semantic processing, sentence processing, and verbal fluency. Studies of patients with cerebellar damage converge to show that the cerebellum supports sentence processing and verbal fluency. However, to date there are no patient studies that investigated the specific importance of the right posterior cerebellum in language processing, because: (i) case studies presented patients with lesions affecting the anterior cerebellum (with or without damage to the posterior cerebellum), and (ii) group studies combined patients with lesions to different cerebellar regions, without specifically reporting the effects of right posterior cerebellar damage. Here we investigated whether damage to the right posterior cerebellum is critical for sentence processing and verbal fluency in four patients with focal stroke damage to different parts of the right posterior cerebellum (all involving Crus II, and lobules VII and VIII). We examined detailed lesion location by going beyond common anatomical definitions of cerebellar anatomy (i.e., according to lobules or vascular territory), and employed a recently proposed functional parcellation of the cerebellum. All four patients experienced language difficulties that persisted for at least a month after stroke but three performed in the normal range within a year. In contrast, one patient with more damage to lobule IX than the other patients had profound long-lasting impairments in the comprehension and repetition of sentences, and the production of spoken sentences during picture description. Spoken and written word comprehension and visual recognition memory were also impaired, however, verbal fluency was within the normal range, together with object naming, visual perception and verbal short-term memory. This is the first study to show that focal damage to the right posterior cerebellum leads to language difficulties after stroke; and that processing impairments persisted in the case with most damage to lobule IX. We discuss these results in relation to current theories of cerebellar contribution to language processing. Overall, our study highlights the need for longitudinal studies of language function in patients with focal damage to different cerebellar regions, with functional imaging to understand the mechanisms that support recovery.

## Introduction

We investigate whether the right posterior cerebellum is critical for language processing, as suggested by imaging studies of healthy adults. To this end, we present results of standardized language tests for four patients who all have a focal unilateral lesion affecting different parts of the right posterior cerebellum.

By way of general background, it is now well accepted that the cerebellum is important for higher cognitive functions in addition to motor processing (Argyropoulos et al., 2020), with many studies highlighting its importance for language (Leiner et al., 1986; De Smet et al., 2013; Schmahmann, 2019). Anterior cerebellar lobules I-V, together with adjacent parts of lobule VI, have been typically associated with motor processing, including articulation (Nota and Honda, 2004; Ackermann et al., 2007; Stoodley and Schmahmann, 2010; Correia et al., 2020), while cognitive processing is more commonly associated with the posterior cerebellar lobules VI through IX (Stoodley and Schmahmann, 2010).

Pertinent to the current study, functional imaging studies of healthy adults have documented right lateralized language related activation in posterior cerebellum during verb generation (Frings et al., 2006; Spencer et al., 2007; Stoodley et al., 2010), semantic processing (Petersen et al., 1989; Xiang et al., 2003), and sentence processing (Stowe et al., 2004; Strelnikov et al., 2006; D’Mello et al., 2017; Guell et al., 2018b), with meta-analyses showing that the right posterior cerebellum is activated by a range of language tasks (Stoodley and Schmahmann, 2009; Keren Happuch et al., 2014). Further research by King et al. (2019), using an extensive behavioral battery, divided the cerebellum into distinct functional regions whose boundaries did not coincide with those defined anatomically. Two large functional regions within the right posterior cerebellum (regions 8 and 9, ibid.) were defined as primarily “language regions.” The rest of the posterior cerebellum was associated with divided attention, autobiographical recall, and action observation, among other functions.

Taken together, findings to date suggest that (parts of) the right posterior cerebellum play an important role in language processing. However, determining whether the right posterior cerebellum is necessary for language function requires behavioral data from brain stimulation (either non-invasive; Walsh and Cowey, 1998; or direct; Lurito et al., 2000; Duffau, 2008), or from participants who have focal brain damage (Price et al., 1999).

Stimulation studies have confirmed the critical role of the right cerebellum in sentence processing, specifically, in generating feed forward predictions (Stowe et al., 2004; Moberget et al., 2014; Lesage et al., 2017; Mariën and Borgatti, 2018). In one study, the commonly seen effect in which participants respond faster to predictable trials compared with unpredictable trials, was enhanced by anodal transcranial direct current stimulation(tDCS) to the right cerebellum (Miall et al., 2016). Likewise, cathodal tDCS (Miall et al., 2016) or repetitive transcranial magnetic stimulation (rTMS; Lesage et al., 2012) at the same site, and using the same task, had the opposite behavioral effect. Additionally, TMS studies found an effect of stimulation of the right posterior cerebellum on semantic tasks performance, but only under specific conditions. One study found that the effect on a primed lexical decision task was specific to site (seen after stimulation to lateral Crus I but not medial lobules VI, VII), and task (participants’ performance was enhanced only when the priming involved semantic associative noun-to-verb pairs; Argyropoulos and Muggleton, 2013). In another study, stimulation impaired performance on semantic matching of written words only for matching, but not for non-matching pairs (Gatti et al., 2020). Critically, however, fine-grained anatomical distinction is difficult to achieve in stimulation studies of the cerebellum.

Studies of patients with cerebellar damage in various sites most consistently report impairments in sentence processing and verbal fluency (reviewed in Silveri, 2020). However, the effects of focal stroke are not established.

Early case studies associated sentence processing impairments with right cerebellar stroke (Silveri et al., 1994; Zettin et al., 1997; Gasparini et al., 1999; Marien et al., 2000). This finding was supported by a large group study by Leggio et al. (2008) who found that patients with right cerebellar damage had impairments in sequencing verbal material (written sentences) but not pictures of scenes or spatial material, while patients with left cerebellar lesions showed the opposite effect. However, left cerebellar strokes have also been associated with sentence processing impairments in case and group studies (Cook et al., 2004; Murdoch and Whelan, 2007), and others found no differences in sentence processing for left and right cerebellar strokes (Justus, 2004; Karaci et al., 2008).

Impaired verbal fluency has also been reported in patients with left or right cerebellar damage (Leggio, 2000; Neau et al., 2000; Murdoch and Whelan, 2007; Peterburs et al., 2010; Schweizer et al., 2010; Alexander et al., 2012), with some studies suggesting that phonemic fluency is more affected than semantic fluency (Leggio, 2000; Murdoch and Whelan, 2007). Using lesion-symptom mapping in 21 patients with left and right cerebellar damage, Richter et al. (2007) located the most significant association between verbal fluency impairments and cerebellar damage to right Crus II (in the posterior cerebellum).

While patient studies converge to show that the cerebellum is critical for language processing, they do not provide evidence that the right posterior cerebellum is a critical site, due to the following caveats: (i) some studies included patients with non-focal damage (Marien et al., 1996; Fabbro et al., 2004); (ii) others included patients with large lesions, affecting multiple functional cerebellar regions (Silveri et al., 1994; Zettin et al., 1997); (iii) still others did not give precise details on the location of the lesion (Cook et al., 2004). Even when patients have isolated and well defined lesions, the role of the right posterior cerebellum in language still cannot be determined as (iv) case studies present patients with lesions affecting anterior parts of the cerebellum, with or without damage to the posterior lobes (Silveri et al., 1994; Marien et al., 1996, 2000; Zettin et al., 1997; Gasparini et al., 1999); and (v) group studies include patients with varied lesion locations, reporting language performance at the group level only (Justus, 2004; Murdoch and Whelan, 2007; Karaci et al., 2008).

Here we aimed to determine if the right posterior cerebellum is critical for language function. In order to address caveats of previous studies, we (i) examined four case studies of patients with isolated unilateral right posterior cerebellar lesions; (ii) focused on sentence processing and verbal fluency, the two language functions most commonly associated with cerebellar damage; and (iii) examined lesion location by going beyond common anatomical (e.g., Crus II) and vascular territory (e.g., PICA) definitions of lesion location, and employing a recently published functional parcellation of the cerebellum (King et al., 2019).

## Materials and Methods

### Participants

**FIGURE 1:**
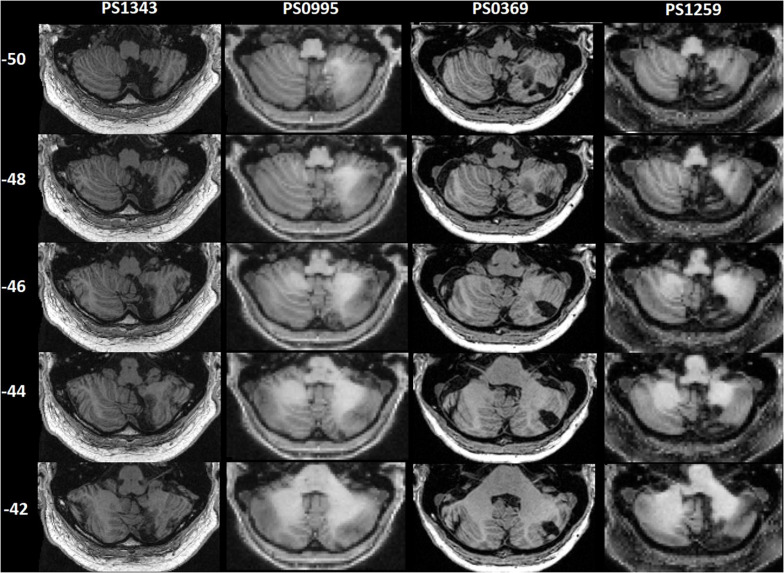
Patients’ lesions displayed on axial slices. Patient IDs are shown above the images. z coordinates in MNI space are shown on the left.

Patients were selected from the Predicting Language Outcome and Recovery After Stroke (PLORAS) database that records behavioral, demographic and imaging data from participants with a history of adult stroke as defined by a neurologist (Seghier et al., 2016). At the time of study, our database included ∼ 1,000 patients who: (i) were raised using English as their native language; (ii) were right handed prior to their stroke; and (iii) had no history of concomitant neurological or psychiatric illness. All participants gave written informed consent according to the Declaration of Helsinki prior to being included in the study, and were compensated financially for their time in accordance with the London Queen Square Research Ethics Committee (study code: 13/LO/1515 and 19/LO/1755). We searched the database for patients with unilateral focal lesions affecting the right posterior cerebellum. This search was first done according to the neurologist definition of the site of damage, which labels every patient for presence or absence of damage in each lobe or region (frontal, temporal, cerebellar, etc.), and hemisphere (right or left). We then examined the detailed neurologist lesion description and the MRI scan, to find those patients whose lesion affected the right posterior cerebellum. Focal lesion was defined as damage affecting the right posterior cerebellum, without additional damage to the left cerebellum, anterior cerebellar lobes or supratentorial regions. Four patients fitted this inclusion criteria, and all patients experienced language difficulties acutely, according to their clinical notes and/or self-report. These included difficulties with verbal short term memory and word finding. All patients further reported having some difficulty with speech and language at 1 month post-stroke. Demographic and clinical details of the patients, and information about Speech and Language Therapy, are given in Table 1. Lesions are presented in Figure 1.

**TABLE 1:** Demographic and lesion information of patients.

Participant (PLORAS ID)	PS1343	PS0995	PS0369	PS1259
Lesion location	Crus II, VIIb, VIIIa/b, IX	Crus II, VIIb, VIIIb	Crus II, VIIb, VIIIa/b, IX	Crus II, VIIa, VIIIa/b
Number of years of formal education	16	18	12	17
Age at Stroke (years)	44.6	31.6	31.1	54.2
Age at test (years)	49.1	32.6	40.3	56.1
Time between stroke and test (months)	54	11	110	23
Gender	M	F	F	M
Speech and language therapy	Comprehension, long- and short-term memory, one session a week for under a year	No SLT given	Therapy given only while in hospital (first 6 weeks)	Unknown
Occupation	Information technology	Administration	Unknown	Retired stroke nurse then a business consultant

### Acquisition and Processing of MRI Data

Three different MRI scanners (Siemens Healthcare, Erlangen, Germany) were used to acquire T1-weighted structural images, each with 176 sagittal slices, a matrix size of 256 × 224 and a final spatial resolution of 1 mm isotropic voxels. Patient PS0369 was scanned on a 1.5T Sonata scanner (repetition time (TR)/echo time (TE)/inversion time (TI) = 12.24/3.56/530 ms), patients PS1259 and PS0995 were scanned on a 3T Trio scanner (TR/TE/TI = 7.92/2.48/910 ms), and patient PS1343 was scanned on a 3T PRISMA scanner (TR/TE/TI = 2,530/3.34/1,100 ms). The T1-weighted structural images were acquired using an optimized 3D modified driven equilibrium Fourier transform (MDEFT) sequence for the Trio and Sonata scanners, and using a 3D magnetization-prepared rapid acquisition gradient-echo (MPRAGE) sequence for the PRISMA scanner.

Data pre-processing was performed in the Statistical Parametric Mapping software (SPM12; Wellcome Centre for Human Neuroimaging, London, United Kingdom;^1^), running in MATLAB environment (2018a Mathworks, Sherbon, MA, United States). As our automated lesion identification toolbox (Seghier et al., 2008) did not recognize some of the lesions of the four participants, their lesions were manually delineated by one author (SG). The lesions were identified on a slice by slice basis on the T1-weighted images in the native space using MRIcron software^2^. The outline of the lesion was drawn on the outer borders of abnormally intense regions. Abnormal tissue was included in the lesion definition when there was a clear asymmetry between the lesioned and non-lesioned hemisphere, excluding areas of abnormality far from the lesion that could be related to mass effects. Periventricular regions were defined as lesioned only when there was a clear signal intensity change in the area and the lesion extended all the way to the periventricular space. Areas surrounding enlarged ventricles with normal signal intensity, or white matter changes appearing on both hemispheres, were not defined as lesioned. T1-weighted images were spatially normalized using the unified segmentation-normalization procedure, which segments, bias corrects and spatially normalizes the images in the same model. Deformation field parameters were then applied to the binary lesion images, and the output was inspected by eye.

### Neuropsychological Assessment

Participants completed the Comprehensive Aphasia Test (CAT; Swinburn et al., 2004) which consists of 27 subscales measuring various language functions, and a cognitive screen. The CAT defines behavioral T-scores for each task, with lower T-scores indicating poorer performance. Each score defines how a given patient performed relative to a distribution of 60 patients with post-stroke aphasia. The threshold of impairment in each task is derived from a second population of 27 people with normal language and cognition, and it varies with task (for cut-off scores and maximum possible scores on each task, see Table 2). Performance below a cut-off score implies that the patient would be in the bottom 5% of the neurologically intact population. Note that patients with aphasia can score within the normal range on some subtests of the CAT; this occurs most frequently when patients have relatively mild aphasia or when the subtests are relatively easy (Howard et al., 2010). Further details on the scoring procedure and standards are given in the CAT manual (Swinburn et al., 2004). The prime focus of the current analysis was the verbal fluency task (semantic and phonemic), and tasks requiring sentence processing (spoken and written, comprehension and production).

**TABLE 2:** Comprehensive aphasia test t-scores obtained by patients.

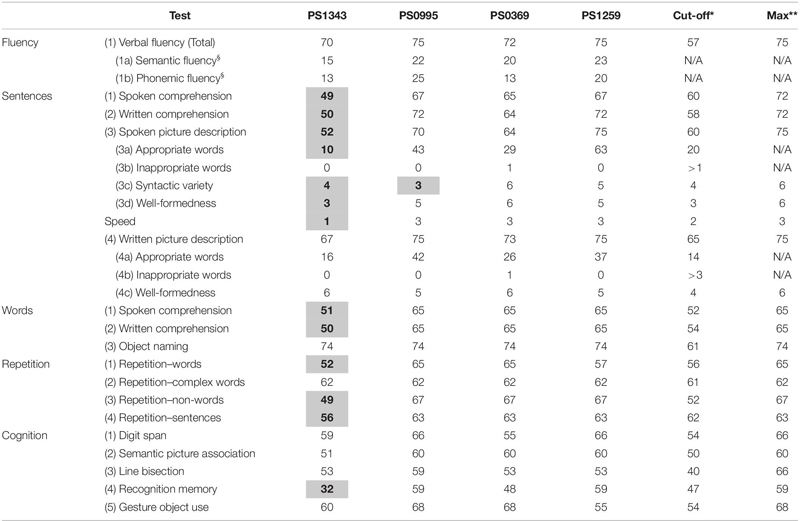

Impaired Scores are in bold and highlighted in gray. *Cut-off scores are the highest scores within the impaired range. **Max scores are the highest scores that can be obtained on the test. ^§^ We provide raw scores for verbal fluency sub-tests, as T-scores are not available in the CAT manual.

### Verbal Fluency

Participants performed two tasks: in the semantic fluency task they were given 60 s to say as many words as they could according to a semantic prompt (“Name as many animals as you can”); in the phonemic fluency task, they were given 60 s to say as many words as possible given a phonological prompt (“Name words beginning with the letter ‘s”’). Participants are allowed to make articulatory errors but repeated items (perseverations) are not counted. We provide raw scores for the semantic and phonemic fluency tasks, and a combined T-score for the sum of these two tasks.

### Sentence Processing

#### Comprehension of Spoken and Written Sentences

Scores for sentence comprehension are based on two points per trial for immediate correct responses; one point per trial for correct responses after a self-correction/delay (>5 s)/repetition of stimuli by the examiner; and zero points for trials with incorrect responses. For the spoken sentence comprehension task, participants are presented with 16 spoken sentences of increasing syntactic complexity and four line drawings. They are asked to point to the corresponding target drawing. The written sentence comprehension task is similar to the spoken sentence comprehension, only that sentences are written.

#### Spoken Picture Description

Participants are asked to describe a picture that shows a complex scene. Verbal description produced within the first 1 min is transcribed and scored, using the following five sub scores:

(i) Appropriate information-carrying words: words/word-units that add to the information being conveyed in the correct context. Dysarthric distortions are not penalized. Producing <21 words is considered impaired.
(ii) Inappropriate information-carrying words: each word incorrectly selected (e.g., verbal paraphasias, neologisms, semantically related/unrelated words) receives one point. Producing more than one inappropriate word is considered impaired.
(iii) Syntactic variety: range of syntactic structures used (e.g., wide range of verbs, pronouns, use of embedded clauses). This is scored on a 0–6 scale, ranging from 0 = no/stereotypic syntactic structures, to 6 = full, or nearly full, range of syntactic structures. Scores of 0–3 are considered impaired.
(iv) Grammatical well-formedness: the degree of grammatical accuracy entailed in the sentences. Errors can include (but are not restricted to): omissions of arguments following transitive verbs, inappropriate verb tense or number marking, or incorrect inflections or auxiliaries. This is scored on a 0–6 scale, ranging from 0 = no phrases well-formed, to 6 = all phrases well-formed, no phrases omitted. Scores of 0–4 are considered impaired.
(v) Speed: scored on a 0–3 scale, with 0 = significant and consistent delay; and 3 = normal speed of speech production. Scores of 0–2 are considered impaired.

#### Written Picture Description

This task is similar to the spoken picture description task, except that the participants are asked to provide a written description of what is happening in the same picture. Written description given within the first 3 min is scored as above, using the following three sub-scores: (i) Appropriate information carrying words (producing <15 words is considered impaired); (ii) Inappropriate information carrying words (producing more than three inappropriate words is considered impaired); and (iii) Grammatical well-formedness (scores of 0–4 are considered impaired).

### Word Processing

We examined tasks requiring single word processing in order to evaluate whether some of the documented sentence level impairments can be attributed to word-level impairments. In these tasks, each trial is given 0/1/2 points, based on the same criteria as for the sentence comprehension tasks.

#### Comprehension of Spoken and Written Words

These tasks are similar to the spoken and written sentence comprehension tasks, with the difference that single words are presented, and alongside the target drawing, the three distractors include: one which is phonologically related to the target, one semantically related and one unrelated. There are 15 trials in each task.

#### Production and Repetition of Spoken Words

For word production, line drawings of 24 objects (e.g., knife) are presented one at a time, with instructions to name them aloud. In the three repetition tasks, participants are presented with 16 spoken words (e.g., “plant”), three complex words (e.g., “defrosted”), and five non-words (e.g., “trimpy”), one at a time, for immediate repetition. Articulatory errors (e.g., dysarthric distortions) not affecting the perceptual identity of the target are scored as correct. Verbal, phonemic, neologistic and dyspraxic errors are scored as incorrect.

### Verbal Short-Term Memory

As studies suggest that some difficulties in sentence processing can be attributed to a deficient verbal short-term memory (Warrington and Shallice, 1969; Warrington et al., 1971; Saffran and Marin, 1975; Vallar et al., 1997), we assessed verbal short-term memory using the sentence repetition and digit span tasks. In these tasks participants are presented with sentences or digit strings of increasing length, for immediate repetition. There are two trials of each length. Participants only have to repeat one sentence / digit string of each length, unless they repeated it incorrectly. Sentence length ranges from 3 to 6 content words. Digit strings start with two digits and build up to seven digits. Phonemic errors, apraxic errors and dysarthic distortions are accepted as correct, as the aim of the tasks is to assess memory span only.

### Assessment of Apraxia

Apraxia of speech was assessed based on the Apraxia Battery for Adults, 2nd edition (ABA-II; Dabul, 1979). Using the “Inventory of articulation characteristics of apraxia” (subtest 6, ibid) we coded each patient’s spoken picture description from the CAT. The scale records 15 behaviors associated with speech production, including various types of errors (e.g., phonemic anticipatory, perseverative, transposition, voicing, or vowel errors), visible/audible searching or off-target attempts at the word, difficulty with speech initiation, abnormal prosodic features, awareness of errors and ability to correct them, among others. A score of 5 and above is indicative of apraxia.

### Anatomical and Functional Definition of the Lesion Site

For the definition of cerebellar “functional regions,” we employed the multi-domain task battery (MDTB) based parcellation by King et al. (2019), which divides the entire cerebellum into 10 functional regions. Anatomical parcellation into lobes was based on the Spatially Unbiased Atlas Template of the Cerebellum and Brainstem (SUIT^3^; Diedrichsen, 2006). We calculated the percent of overlap between the manually delineated binary lesion of each patient, and each functional / anatomical region.

## Results

Patients’ scores on each task are reported in Table 2. All patients had normal verbal fluency and all but one (PS1343) had normal sentence processing, across a range of tasks. In the assessment of apraxia, PS1259 had a score of 1 and the others had a score of 0. Hence, none of the patients were classified as having apraxia, according to the ABA-II. Below we focus on understanding the impaired performance observed in PS1343 only.

### Sentence Processing

PS1343 had impaired sentence processing across modalities (spoken and written) and domains (production and comprehension). Both spoken and written sentence comprehension were impaired, with the patient making two errors in each task, with further penalty for delays.

Sentence repetition was impaired due to verb and noun replacements. Spoken sentence production during picture description was impaired due to the low number of content words produced, limited syntactic variety, some grammatical errors (e.g., “cat hungry for fish,” “Book dropping on man”) and low production speed. In contrast, spontaneous speech production was noted to be fluent, and verbose. Written picture description was in the very low end of the normal range. See Supplementary Material for transcripts of sentence repetition responses and the spoken picture descriptions.

### Word Processing

PS1343 had impaired word comprehension across modalities (spoken and written), making one semantic error in the spoken modality (selected “cow” when hearing “bull”) and having delayed responses in both modalities. Word and non-word repetition were also impaired, with phonemic errors, but object naming was within the normal range, as was reading aloud words and non-words.

### Other Cognitive Abilities

PS1343 had impaired visual recognition memory according to the recognition memory task. This was at the level of memory rather than perception because visual recognition was intact according to: semantic association of pictures, object naming and gesturing the use of objects. Likewise, although word, non-word and sentence repetition were all impaired, this could not be explained by impaired verbal short-term memory, as this was intact according to the digit span task.

### Lesion Location and Demographics

**FIGURE 2:**
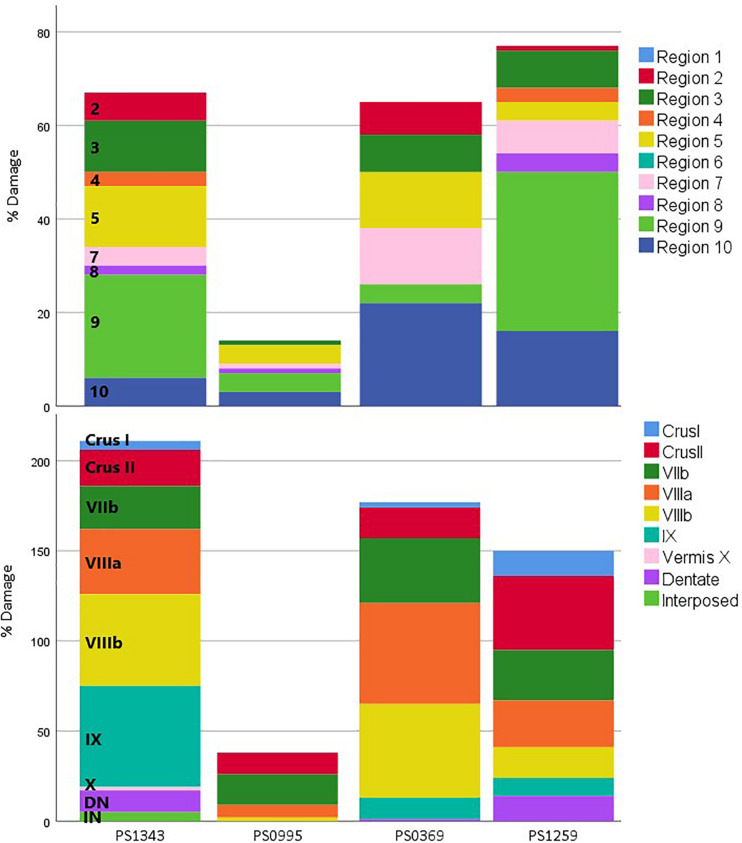
Percent damage to each region for each patient. Percent damage to functional (top; King et al., 2019) and anatomical (bottom; Diedrichsen, 2006) regions. The size of each colored section represents the percent of damage to that region. This allows comparing the damage to each region between the patients. Regions not damaged in any of the patients are not included in this figure. DN, Dentate Nucleus; IN, Interposed Nucleus.

Within our sample of four patients, PS1343 did not have the largest lesion but did have more damage to lobule IX than the other three patients. Additional damage in PS1343 was identified in Crus II, lobule VIIb, VIIIa, and VIIIb–regions that were also damaged in PS0369 (see Figure 2 and Table 3). Looking at the functional regions, PS1343 had substantial damage to language region nine, and additional damage to regions three (saccades, visual working memory, and visual letter recognition) and five (divided attention, mental arithmetic, and active maintenance). While all of these areas were also affected in at least one of the other patients, the lesion distribution differed between the patients (Figure 2 and Table 3).

**TABLE 3:** Percent overlap between functional and anatomical regions, and patients’ lesions.

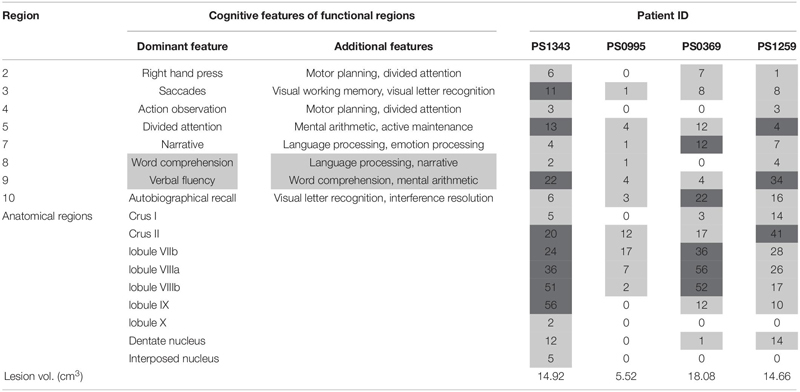

*Numbers represent the percent of damage that each region (anatomical or functional) had incurred. Regions not damaged in any of the patients are not included in the table. Functional regions (King et al., 2019): Dominant and additional features are processes which activate each region. Dominant features weigh more than the additional features. Regions 8 and 9 which are the main language processing regions, are highlighted. Anatomical regions (Diedrichsen, 2006): percent damage for lobules VIIIa, VIIIb, and IX represent the combined damage for the hemispheric and vermal regions. In all cases, damage to the vermal region was < 10%. When the overlap is < 1% it is marked as 0%. For ease of reading, the percent overlap is highlighted by increasing shades of gray, from 0% overlap in white, to > 20% in dark gray.*

We also note that PS1343 was not older, less educated or less chronic than all other three patients (Table 1).

## Discussion

In a study of four patients with focal lesions affecting various parts of the right posterior cerebellum, we found that all four patients had language difficulties early after their stroke but only one patient, with a lesion affecting Crus II and lobules VIIb, VIIIa/b, and IX, had aphasia more than a year after the stroke. This persistent language impairment affected tasks requiring sentence processing but not verbal fluency. In addition, comprehension of spoken and written words, repetition of words and non-words and visual recognition memory were all impaired, while object naming and digit span were within the normal range. Below we discuss these behavioral impairments and how they might relate to the location of stroke damage.

### The Language Impairment Observed in PS1343

PS1343 had impairments on the sentence processing tasks, across domains (comprehension and production) and modalities (spoken and written). These impairments were not specific to sentences because PS1343 also had impairments on the spoken and written word comprehension tasks and on the word and non-word repetition tasks. Nor can the impairments be explained by visual or auditory perceptual problems because PS1343 was not impaired for semantic picture matching or repeating back heard numbers in the digit span task. Speech articulation was also normal, with no dysarthric or dyspraxic errors documented in any of the speech production tasks.

Taken together, these results suggest that PS1343 had a verbal comprehension impairment that affected the patient’s ability to match spoken or written words and sentences to the corresponding pictures. This is sufficient to explain: (1) the difficulty matching heard and written speech to pictures during the comprehension tasks; and, (2) poor repetition.

A verbal comprehension impairment cannot, however, explain why PS1343 performed poorly on two additional tasks: the delayed visual recognition memory task, which involved remembering which pictures he had seen during the semantic picture association task, and the spoken picture description. We therefore suggest that PS1343 had other processing impairments in addition to the one at the level of verbal comprehension. The visual recognition memory impairment could partially explain why PS1343 struggled to describe the events in a picture, despite having excellent object naming abilities. Specifically, when describing a picture, the patient needs to remember what has been seen and described while searching for new events to describe. PS1343 struggled with this task, only producing 10 words in a minute, which contributed to the low syntactic variety score. However, it cannot fully explain the grammatical errors seen in the picture description which might reflect grammatical deficits commonly seen following right cerebellar damage. Lastly, the scores obtained on the written picture description task were very low but within the normal range, suggesting he is not impaired in picture description across all modalities. Writing can support the descriptive task in a number of different ways and so the reason why the scores on the written version of the task might be unimpaired are uncertain.

### Language Recovery in PS0995, PS1259, and PS0369

The three other patients with right posterior cerebellar damage (PS0995, PS1259, and PS0369) reported impaired speech and language early after their stroke, but each performed in the normal range on our language assessments administered after a year. Although this does not exclude the possibility that these patients have subtle language impairments that were not detected by our assessments (Mariën et al., 2014; Guell et al., 2015), their performance is strikingly different to the persistent and severe impairments we report above for PS1343. One explanation for the inter-patient variability is that the patients had different premorbid functional anatomy and cerebellar reserve (Mitoma et al., 2020) which influenced the initial level of impairment, and/or the potential for recovery over time. Demonstrating this requires future longitudinal studies that trace the trajectory of recovery, and changes in neural processing, in patients who have damage to the same cerebellar regions.

A second explanation of inter-patient variability is that PS1343 (with the language impairment) had damage to parts of the cerebellum that were not damaged in the other three patients (without language impairments). To investigate this, we defined the anatomy of the lesions based on a standard anatomical parcellation, as well as a recently published functional parcellation, and neither of these methods by itself could clearly explain the observed variability in performance, across our four patients. For example, the sustained language impairment in PS1343 cannot be solely explained by damage to the language regions defined by King et al. (2019), as the lesions of other patients (e.g., PS1259) affected these areas as well and to a similar degree, but did not result in a sustained language deficit. Additionally, PS1343 had damage to multiple functional divisions in the right posterior cerebellum, including motor and non-motor areas, and hence we cannot determine which part, or combination of parts was responsible for the patient’s impairments. This will require further studies of patients with differing degrees of damage to different posterior cerebellar regions. However, the distinct feature in PS1343’s brain scan that might have explained the language deficit was that, unlike the others, he had substantial damage to lobule IX, in addition to damage to Crus II, and lobules VII and VIII. We now turn to discuss the role of lobule IX in language processing.

### The Role of Right Cerebellar Lobule IX in Language Processing

The role of lobule IX in language processing has been inconsistently reported. In fMRI studies, this could reflect false negative results when a limited field of view excludes lobule IX in the most inferior portion of the cerebellum (Habas et al., 2009). Positive evidence that lobule IX contributes to language processing comes from several sources. For example, a lesion-symptom mapping study of 18 patients with cerebellar damage associated a large right lateralized region extending from Crus I/II to lobule IX with impairments in picture naming, but not with deficits in motor, executive, or visuo-spatial abilities (Stoodley et al., 2016). Analyses of the Human Connectome Project data (Van Essen et al., 2013), revealed activation in right lobule IX associated with story comprehension (Diedrichsen and Zotow, 2015; Guell et al., 2018a) and Guell et al. (2018a) refer to this area as the third non-motor representation of the cerebellum, which is active during cognitive tasks together with the first (lobule VI/Crus I) and second (lobule VIII) non-motor areas. According to King et al. (2019) most of lobule IX is a language region (Region 8), in addition to the language processing in Crus I and II, but the authors do not suggest that there is a functional distinction between Region 8 in Crus I/II and that of lobule IX.

Interestingly, a role for bilateral lobule IX in more general cognitive processing has been suggested by findings showing that lobule IX forms part of the Default Mode Network (DMN) in healthy adults (Habas et al., 2009; Buckner et al., 2011; Dobromyslin et al., 2012; Stephen et al., 2018), and in stroke patients (Li et al., 2013). Patients with subcortical stroke had reduced resting-state functional connectivity between lobule IX and the cortical hubs of the DMN (Li et al., 2013), and reduced integration between undamaged hubs of the DMN has been associated with poorer performance on cognitive tasks post-stroke (Dacosta-Aguayo et al., 2015). Integrity of the DMN has been associated with numerous functions, including emotional processing, self-referential mental activity, and recollection (Raichle, 2015), and as such, without future research, it is difficult to ascertain the mechanisms in which damage to the DMN contributed to the complex cognitive profile seen in PS1343. Future studies using functional connectivity analyses might usefully identify changes in the DMN network and their relation to language performance, in patients with lesions to lobule IX.

### Verbal Fluency

All four patients had normal verbal fluency, as tested using semantic and phonemic verbal fluency tasks. This is in line with prior findings that verbal fluency can be intact following cerebellar damage (Beldarrain et al., 1997; Hokkanen et al., 2006), and an fMRI study of verbal fluency (phonemic and semantic) which found cerebellar activation in the right anterior lobe and most lateral portions of Crus I and II (Schlösser et al., 1998), regions which were not damaged in our patient group. However, other studies have documented verbal fluency impairments following cerebellar lesion in chronic stroke patients (Leggio, 2000; Neau et al., 2000; Murdoch and Whelan, 2007; Peterburs et al., 2010; Schweizer et al., 2010; Alexander et al., 2012) and tDCS over the right posterolateral cerebellum significantly improved phonemic fluency in healthy adults (Turkeltaub et al., 2016). The difference between our findings and those of previous studies is unlikely to be related to task administration or scoring, as the verbal fluency task is widely used, and administered and scored in the same way across studies. We therefore suggest that inter-study differences in the documentation of verbal fluency impairments are more likely to be related to the specific lesion location. While studies which reported verbal fluency impairments following right cerebellar damage included patients with posterior lesions, the effects which show an impairment at the group level might be driven by patients with lesions in other parts of the cerebellum. And although some studies have examined inter-patient variability, none of them provide clear evidence that right posterior cerebellar lesions exclusively, or consistently, affect verbal fluency.

### Future Directions and Study Caveats

Our study demonstrated that some patients with right posterior cerebellar damage do not present with symptoms of aphasia, months or years after stroke. This is in line with previous studies which showed that patients with cerebellar damage have fast and often complete recovery (Holmes, 1917; Ackermann et al., 1992; Zettin et al., 1997; Schweizer et al., 2010). Our data regarding patients’ behavioral performance at the acute stage is limited to clinical notes and self-report. Future longitudinal studies, starting at the acute stage after stroke could track the course of recovery among patients with posterior cerebellar damage. If recovery is supported by functional reorganization, then longitudinal functional imaging could shed light on the mechanisms underlying such recovery, and potentially explain the difference between the patients which do, and do not, recover over time. In addition, finer functional distinction between lateral and medial parts of the right posterior cerebellum is still needed, in order to further explain inter-patient variability in recovery of language.

Secondly, using other imaging modalities could uncover possible additional damage outside the patients’ lesions, which cannot be seen on a standard T1-weighted image, and can potentially explain inter-patient variability in behavioral performance. A recent study of patients with chronic aphasia due to left cortical damage demonstrated that 4 weeks of tDCS to the right cerebellum enhanced patients’ performance on verb generation (Marangolo et al., 2018). This finding supports the idea that cerebellar-cortical connections are involved in aphasia symptoms and recovery. And indeed, some patients with cerebellar lesion show contra-lateral cortical hypoperfusion (Broich et al., 1987; Boni et al., 1992; Marien et al., 1996; Beldarrain et al., 1997), while others display microstructural abnormalities in cerebellar efferent and afferent white matter tracts (measured using Diffusion Tensor Imaging, Olivito et al., 2017). Both of these abnormalities have been associated with symptoms of aphasia (Hillis et al., 2002; Kümmerer et al., 2013).

Third, our group sample size was small, as in other studies of patients with focal cerebellar strokes (Fabbro et al., 2000; Marien et al., 2000). It was not possible to increase our sample size because only 4 patients in a database of more than 1,000 stroke survivors met our inclusion criteria. This can be explained by (i) the rarity of cerebellar strokes (∼2% of total strokes according to Tohgi et al., 1993); (ii) the disproportionally high mortality rate associated with cerebellar stroke (Macdonell et al., 1987; Tohgi et al., 1993; Nickel et al., 2018); (iii) the more subtle manifestation of clinical symptoms among cerebellar stroke survivors (Macdonell et al., 1987; Tohgi et al., 1993; Nickel et al., 2018); and, (iv) our search for a specific lesion site among those with cerebellar stroke.

Lastly, in this study we focused on language impairments, but we cannot rule out that the patients have other symptoms accompanying these impairments, such as abnormal executive function, visuospatial cognition, and affect, therefore allowing a diagnosis of Cerebellar Cognitive Affective Syndrome (CCAS; Schmahmann and Sherman, 1998; Argyropoulos et al., 2020). Co-occurrence of impairments in other cognitive and affective domains, and a potential diagnosis of CCAS in the patients studied here or those with similarly focal lesions, can be the focus of future studies.

## Conclusion

The present study sheds light on the role of the right posterior cerebellum in language processing after stroke. We found that one of our four patients with focal damage to the right posterior cerebellum suffers from persistent language difficulties, affecting verbal comprehension and production, and auditory repetition, but sparing verbal fluency, object naming and digit span. Although it cannot be ruled out that premorbid differences underlie the behavioral variability in these patients, the analyses of the lesion with regard to its location within established functional and anatomical parcellations suggest that the additional damage to lobule IX in the patient with aphasia could explain some of the observed deficits. Our study has implications for future research into the cerebellar mechanisms that support language processing in three directions. First, it highlights the importance of providing finer analysis of lesion location of patients, both in relation to other patients’ lesions, and to common functional and anatomical parcellations. Second, it motivates functional imaging experiments to understand how patients recover from language impairments after focal cerebellar damage, and third, the functional importance of different parts of the right posterior cerebellum to language processing could be investigated with non-invasive neurostimulation techniques.

## Data Availability Statement

The raw data supporting the conclusions of this article will be made available by the authors, without undue reservation.

## Ethics Statement

The studies involving human participants were reviewed and approved by the London Queen Square Research Ethics Committee. The patients/participants provided their written informed consent to participate in this study.

## Author Contributions

SG, LS, and CP developed the research ideas. SG and LS developed hypotheses, conducted analyses, interpreted data, and drafted the manuscript. SR coded, analysed data, and revised drafts of the manuscript. CP and DG revised drafts of the manuscript, contributed in discussing analyses, and provided critical revisions of the manuscript. CP designed the data collection protocol. All authors approved the submitted version.

## Conflict of Interest

The authors declare that the research was conducted in the absence of any commercial or financial relationships that could be construed as a potential conflict of interest.

## Footnotes

1 https://www.fil.ion.ucl.ac.uk/spm/

2 http://www.mccauslandcenter.sc.edu/mricro/

3 http://www.diedrichsenlab.org/imaging/suit.htm
