# Supplementary material for: The Effect of Focal Damage to the Right Medial Posterior Cerebellum on Word and Sentence Comprehension and Production
Source: Front Hum Neurosci. 2021 May 20;15:664650. doi: 10.3389/fnhum.2021.664650 (PMC8172582; doi:10.3389/fnhum.2021.664650)
Supplement: Supplementary file 1 [file Table_1.DOCX]

Supplementary Material

# Sentence Repetition

## PS1343

The children listened as the man [correct: teacher] read the story

The boy and girl went up [correct: climbed] the hill and admired the view

It was a long time before the area was made [correct: pronounced] safe

# Spoken Picture Description

## PS1343

A baby playing

ah cat hungry for fish

Book dropping on man

Man asleep

[Prompt from Examiner]

[1 min]

## PS1259

This is a picture of a lounge where there’s a man sat, reli… reclining in a seat with his feet up on the table, resting on some magazines or papers

On the table there’s a cup

Underneath the table there’s a book on the shelf

the child playing with a car on the floor, waving to the man, ahm presumably, because the cat on the shelf is trying to get to the fish, has knocked the books over, one of which is about to hit the man, which is why the child is waving

so, on the shelf there’s a plant

And the cat has got his paw in the fishbowl, which looks like it has three fish and some shingle or something at the bottom of it

On the bottom shelf there’s an old fashioned hi fi machine with two speakers, presumably stereo, and what looks like, on the left, a set of ah… cassettes, you know, and a XXX and a wide set of CDs

So presumably it plays CDs and cassettes

[1 min]
